# Progress and prospects for blood-stage malaria vaccines

**DOI:** 10.1586/14760584.2016.1141680

**Published:** 2016-02-03

**Authors:** Kazutoyo Miura

**Affiliations:** ^a^ Laboratory of Malaria and Vector Research, National Institute of Allergy and Infectious Diseases, National Institutes of Health, Rockville, MD, USA

**Keywords:** Malaria, *Plasmodium falciparum*, blood-stage, vaccine, in vitro assays, challenge models, clinical trial

## Abstract

There have been significant decreases in malaria mortality and morbidity in the last 10-15 years, and the most advanced pre-erythrocytic malaria vaccine, RTS,S, received a positive opinion from European regulators in July 2015. However, no blood-stage vaccine has reached a phase III trial. The first part of this review summarizes the pros and cons of various assays and models that have been and will be used to predict the efficacy of blood-stage vaccines. In the second part, blood-stage vaccine candidates that showed some efficacy in human clinical trials or controlled human malaria infection models are discussed. Then, candidates under clinical investigation are described in the third part, and other novel candidates and strategies are reviewed in the last part.

There have been significant decreases in malaria mortality and morbidity in the last 10–15 years, and WHO estimated a 47% reduction in mortality between 2000 and 2013 [1]. Many malaria control measures, such as insecticide-treated bed nets (ITNs), indoor residual spraying (IRS), and treatment with artemisinin-based combination therapy (ACT), have contributed to this great achievement. In Africa, where the most virulent human malaria parasites, *Plasmodium falciparum*, still killed ~530,000 people (mainly children under 5 years old) in 2013, it is estimated that scale-up usage of ITNs made the biggest contribution to the reduction (68%), followed by ACT (19%) and IRS (13%) [2]. However, the emergence of mosquitoes and parasites resistant to existing control strategies has increased apprehension about future directions [2,3].

In July 2015, the most advanced malaria vaccine, RTS,S made by GlaxoSmithKline (GSK), received a positive opinion from European regulators for the first time [4]. The RTS,S vaccine is a pre-erythrocytic stage vaccine which is designed to prevent malaria infection and contains part of the circumsporozoite protein (CSP). The 3-year phase III efficacy study, which involved 8922 children (5–17 months old at enrollment) and 6537 infants (6–12 weeks), has shown 36.3% (95% confidence interval (CI): 31.8–40.5%) vaccine efficacy in children against clinical malaria and 25.9% (95% CI: 19.9–31.5%) in infants [5]. This major milestone in malaria vaccine development history has proved that an efficacious malaria vaccine is achievable. However, a more efficacious second-generation vaccine is needed and the duration of efficacy of the current RTS,S vaccine is concerning [6].

The feasibility of blood-stage vaccines has been supported by many epidemiological studies; people living in malaria endemic areas can acquire immunity against severe malaria initially, then clinical malaria [7]. Two passive IgG transfer studies in humans directly established that the immunity is at least in part mediated by antibodies. In the first passive transfer study conducted in The Gambia, children with acute malaria received purified IgGs from Gambian malaria-immune adults [8]. The parasite density dropped significantly from 10,000–230,000 parasites/μl to zero in 8 out of 12 children (the maximum of 80/μl in one child) by day 9, while transfusion of non-IgG fraction of the sera or IgG from malaria naive UK people had no effect. In the second study, IgG from African adults was inoculated to Thai patients with 4200–9000 parasites/μl [9]. The parasitemia went down to 8–90 parasites/μl between 33 and 113 h after the initial inoculation. The Thai study has shown that IgG from a different geographical location has the capacity to kill parasites *in vivo*. The mechanism of parasite killing by the antibodies has not yet been resolved, but if a blood-stage vaccine can elicit such effective antibodies in humans, the vaccine is likely to prevent clinical malaria. The Malaria Vaccine Technology Roadmap updated in November 2013 targets two strategic goals by 2030 [10]: (1) vaccines with >75% efficacy against clinical malaria and (2) vaccines that reduce transmission of the parasite and thereby substantially reduce malaria infection. A new blood-stage vaccine or a combination of vaccines against the blood-stage and pre-erythrocytic stages of malaria is needed to achieve the 75% goal. If the vaccine has a strong enough efficacy, it can also reduce transmission by significantly lessening the gametocyte numbers in humans.

The first part of this review summarizes the pros and cons of various assays and models which have been and will be used to predict efficacy of blood-stage vaccines. In the second part, blood-stage vaccine candidates which showed some efficacy in human clinical trials or controlled human malaria infection (CHMI) models are discussed. Then other candidates under clinical investigation are described in the third part, and novel candidates and strategies, which are not mentioned in the first three, are reviewed in the last part. This manuscript does not cover vaccines against pregnancy malaria or *Plasmodium vivax* vaccines since they are discussed elsewhere [11,12].

## How to evaluate vaccine candidates

It is well acknowledged that developing a successful vaccine takes a long time and a great deal of money. In case of RTS,S, GSK initiated the development of this vaccine in the late 1980s, and GSK and the Bill and Melinda Gates Foundation have invested approximately $610 million to date [13]. Therefore, it is very important to establish a surrogate assay(s) and/or model(s), by which we can down-select or terminate an unsuccessful vaccine as soon as possible. By doing that we can focus on more promising novel vaccines. However, since none of the blood-stage vaccines have shown a strong efficacy in the field (i.e. either in phase II or III clinical trials), no assay/model can be established as a surrogate. Many assays and models have been utilized during the RTS,S preclinical and clinical studies, but recent data indicate that anti-circumsporozoite antibody titers are the best surrogate of protection based on the phase III study results [6]. At this moment, only a phase II trial is the best ‘surrogate’ assay for testing the efficacy of blood-stage vaccines, but we cannot reach a phase II trial without evaluating the vaccine candidates by some assays/models. Therefore, the following sections discuss pros and cons of each assay which has been (or will be) used for blood-stage vaccine development.

### Enzyme-linked immunosorbent assay (ELISA), western blot, and immunofluorescence assay (IFA)

As described before, two human passive transfer studies clearly showed that antibodies are the principal contributors to anti-blood-stage parasite immunity in the field (either directly, in combination with other cells, or both). Therefore, many longitudinal (prospective) immuno-epidemiology studies have been conducted to find novel vaccine candidates or to add rational support for further development of existing candidates. Total IgG responses, IgG subclasses, and avidity of antibodies (e.g. using ammonium thiocyanate) were also assessed in many studies. ELISA is easy to perform in many laboratories and relatively easy to standardize compared to other biological assays which are described later. Previously only one or a few proteins were examined in a study, but protein microarrays (which can test more than 1000 proteins simultaneously) began to be applied to longitudinal studies [14]. In preclinical and clinical trials, ELISA is almost always performed to determine the immunogenicity of the test vaccines. However, there are several issues that need to be considered. First of all, the ELISA results depend on the quality of the recombinant proteins (or extracted proteins from parasites) used for ELISA. Indeed, there were two phase I trials conducted with PfCP2.9, which is a recombinant fusion protein of merozoite surface protein 1 (MSP1) and apical membrane antigen 1 (AMA1). The vaccine did induce antibody responses in vaccinees measured by ELISA with the vaccine protein, but the antibodies did not recognize parasites by IFA in one study [15] and did not show any activity in a biological assay, the growth inhibition assay (GIA) [15,16]; in contrast, many human trials have shown MSP1- and AMA1-based vaccines can induce functional antibodies as judged by GIA. In this sense, IFA or western blot using native proteins are better than ELISA with recombinant proteins, but it is not assured that IFA/western positive antibodies can recognize antigen expressed in live parasites, and IFA and western blot assays are not as quantitative as ELISA.

The correlations between immune responses measured by ELISA and clinical protection measured in longitudinal studies vary significantly depending on the study sites [17]. The differences could be caused by many factors: protein used for ELISA, ELISA methodology, endemicity, and parasite strains in the particular field site. Similarly the correlations between GIA results and clinical protection are controversial [18]. Therefore, unless an assay has been performed by multiple investigators in multiple field sites, it is questionable whether we can generalize the findings from one longitudinal study. Another point that must be considered to interpret the data from cohort studies is correlation and causality. When an IgG response (or combination of responses measured by any assay) significantly associates with a reduction of clinical malaria risk in a longitudinal study, the data cannot prove causality, only correlation. For example, several studies have shown breadth of responses and combination of IgGs responses are associated with the risk of clinical malaria [19,20]. The breadth and combination of responses might be a better indicator of malaria exposure (therefore such people may have higher titers against ‘protective’ antigens); it does not necessarily mean such antibodies cause this protection. There is no argument that longitudinal studies are extremely valuable to search for a novel potential candidate and a novel functional assay, which could eventually be a surrogate of vaccine-induced clinical protection. However, because of those limitations, I only discuss results from epidemiological studies in the following sections when it is critical.

### GIA/IIA

The GIA or invasion inhibition assay (IIA) is one of the most widely used functional assays in blood-stage vaccine development. In general, parasites are co-cultured with either control or test antibodies, and % inhibition in parasite numbers (parasitemia) after the incubation is calculated. When parasitemia is measured just after merozoite invasion (usually within 20 h of invasion), the assay is called IIA, while when parasitemia is evaluated at a later time point (40–72 h after starting the culture), it is designated as GIA. If the mechanism of action of test antibody is only to prevent invasion of merozoites into uninfected erythrocytes, IIA and GIA should give the same % inhibition results. On the other hand, GIA can also measure the inhibitory effect on intraerythrocytic parasite development, and such a phenomenon was reported in the case of anti-MSP1 antibody [21]. While inhibitions in invasion, in growth, or both represent differences in parasite biology, in vaccine development as an actual ‘IIA’ is often called ‘GIA’ in many publications. In addition, antibodies which are known to block only parasite invasion are tested by GIA, instead of IIA. Therefore, for simplicity, I will use the terminology of ‘GIA’ in the following manuscript. There are many minor variations in GIA. For example, researchers usually use infected erythrocytes with late trophozoite or schizont stage parasites to initiate the assay, but purified merozoites are also used in some studies; determination of the final parasitemia may be done microscopically, by a flow cytometer, or by a parasite-specific enzymatic activity; parasites may be incubated with antibody less than one cycle (20–40 h) or two cycles (~72 h). A study reported that the final results could differ slightly depending on the methods and types of test antibodies [22].

GIA has been routinely performed in many different laboratories in the world, and it is easy to use different strains of parasites to evaluate the impact of polymorphisms in the target antigens. Therefore, these assays have been utilized not only in many animal immunization studies but also in many phase Ia trials, such as AMA1 [23–25], MSP1 [26], and erythrocyte binding antigen (EBA)-175 [27]. The important point is that the vaccines developed by different investigators and tested in different platforms can induce GIA-positive antibodies when the human antibodies were tested at the same or lower concentrations than those seen in their blood.

Despite the wide usage of GIA, there are two key questions remaining for GIA in vaccine development; one is whether GIA is suitable for trials in malaria-exposed populations. The second, more serious question is whether GIA is a useful assay to predict efficacy in the field. In terms of the first question, it is reported that GIA results could change depending on the population immunized. One example was that AMA1-C1 (a mixture of AMA1-FVO and AMA1-3D7 recombinant proteins) adsorbed on Alhydrogel could induce GIA-positive antibodies in US adults [23], but not in Malian adults [28], while elevations of anti-AMA1 antibody titers measured by ELISA were observed in both populations. Another example was FMP2.1 (AMA1-3D7 protein) formulated with AS02A adjuvant. Similar to the AMA1-C1/Alhydrogel vaccine, the FMP2.1/AS02A vaccine increased anti-AMA1 titers regardless of vaccinees, but increases in GIA activity were only observed in malaria-naive adults [24], but not in malaria-immune adults [29]. Another phase Ib study revealed that the AMA1-C1/Alhydrogel vaccine could increase % inhibition in GIA in Malian children, but only in those who had no GIA activity before immunization (like a malaria-naive population) [30]. In addition, children with higher anti-AMA1 titers at baseline have more ‘interfering’ antibodies, which could block GIA activity of affinity-purified human anti-AMA1 antibodies [31], and such ‘interfering’ antibodies were also observed in Malian adults [32]. The ‘interfering’ antibodies were malaria-specific IgGs, but the target antigen(s) has not been identified. Further investigation is required to determine whether the ‘interfering’ antibodies actually diminish the vaccine efficacy in the field, or are just an artificial observation with *in vitro* GIA. In either case, interpretation of GIA results from vaccine trials in malaria-experienced individuals is complicated.

The second question for GIA is more important. As discussed above, ultimate proof or disproof cannot be done until a blood-stage vaccine shows a measurable efficacy in a phase II (or III) trial. However, many vaccine formulations, which can induce measurable GIA-active antibodies in humans, have not shown significant efficacy in either phase II trials or CHMI models. In one CHMI study, there was a significant inverse correlation between parasite multiplication rate (PMR; fold-increase of parasitemia per 48-h cycle) and GIA activity in AMA1 vaccinees (*p* = 0.02, *n* = 6) [33]. However, the significant correlation disappeared when two control volunteers were included in the analysis (*p* = 0.15, *n* = 8). One possible explanation is that GIA is not a surrogate assay, and the other is that the levels of GIA activity reached in human vaccinees were too low to show any efficacy. The latter possibility is partially supported by monkey challenge studies. When *Aotus* monkeys were immunized with AMA1-based vaccines and then challenged with *P. falciparum* parasites, all monkeys who were protected against the challenge showed >70% inhibition in GIA before parasite challenge [34]. Another *Aotus* monkey challenge study with a MSP1-based vaccine showed that all protected monkeys had >80% inhibition in GIA [35]. Since the GIA conditions in those two studies were different, it is difficult to compare the % inhibition values directly, but both studies suggested that higher GIA activities might be required to show protection at least in the monkey challenge models. If it is also true in humans, a much stronger vaccine formulation needs to be developed.

Recently, Boyle *et al* published that some (but not all) IgGs from Kenyan and Papua New Guinea sera showed higher invasion inhibition in the presence of complement [36]. On the other hand, when Malian adults IgGs (*n* = 19) were tested with or without complement, none of the IgGs showed different % inhibition (unpublished data). The difference might be explained by the methods utilized; Boyle’s IIA was done with purified merozoites, and our GIA with infected erythrocytes. The same group previously published that purified merozoite IIA showed higher % inhibitions compared to the regular infected erythrocyte IIA when the same anti-AMA1 mAbs were tested [37]. In any case, the effect of complement in GIA/IIA needs to be investigated further.

### ADCI

Antibody-dependent cellular inhibition (ADCI) assay is an assay to determine parasite-killing effects of soluble factors (including TNF-α) released from human monocytes which are activated by a test antibody [38]. The African adults’ IgG used in the second passive transfer study showed a positive response in ADCI, but not in GIA [39]. Therefore, ADCI is considered as one of the potential surrogate assays. Since test antibodies may directly block the parasite invasion or growth (which can be measured by GIA) and monocytes may release killing factors without antibody stimulation, ADCI results are usually expressed as a specific growth inhibition (SGI) index, which is intended to exclude the inhibitory effect of monocyte alone and antibody alone. MSP2 [40], MSP3 [41], and glutamate-rich protein (GLURP) [42] vaccines induced ADCI-active antibodies in humans, and affinity purified human anti-serine repeat antigen 5 (SERA-5) antibodies showed positive ADCI [43]. Interestingly, all of those antigens have not been reported to induce GIA-active antibodies.

While ADCI assay is potentially a valuable assay for vaccine development, this assay is not easy to perform and it has been very difficult for many laboratories to execute this assay. The barrier in implementing ADCI assay could be partially explained by the heterogeneity of the human monocytes used. Depending on the subset of monocytes characterized by a series of surface markers, such as CD16, CD14, and CCR2, ADCI activities vary significantly [44]. Even when monocytes were collected from the same individual from different days, the SGI changed from 12.2% to 56.5% [45]. While there is a report attempting increase in the throughput of assay [46], so far ADCI with a monocyte cell line has not been successful despite efforts in many laboratories. To utilize this assay more widely, further definition and optimization are required.

### Phagocytosis/opsonization assay

Opsonization and phagocytosis assays have not been widely utilized for antibody samples from clinical trials as yet, but several immuno-epidemiology studies have shown a correlation between phagocytosis activities and reduction in clinical malaria [47,48]. The phagocytosis assays loosely fall into four categories based on the parasites and phagocytic cells: whether the assay is performed with purified merozoites [47,48] or with infected erythrocytes [49,50] and whether THP-1 human monocyte cell line [47,49,50] or primary peripheral blood mononuclear cells [48,51] are used. The expression pattern of the target antigen determines the parasite source (either merozoites or infected erythrocytes) for a phagocytosis assay. The assay with THP-1 cells is considered to provide more reproducible results. However, the assay cannot cover the diversity of mononuclear cells in individuals and THP-1 cells do not express FcγRIII receptors [52].

Since it takes only 30–120 s for merozoites from egress to invasion [53] and merozoites lose their infectivity very rapidly at 37°C [54], whether phagocytosis of infectious live merozoites mediated by vaccine-induced anti-merozoite antibodies has a significant impact *in vivo* is debatable. On the other hand, parasite-derived antigens expressed on the surface of infected erythrocytes are exposed to human effector mechanisms for a much longer time. However, such antigens which are exposed to the human immune system are known to be highly variable and are called variant surface antigens (VSAs). Therefore, developing a cross-reactive VSA-based vaccine is extremely challenging [55]. Even in recent animal immunization studies, VSA-based (more specifically *P. falciparum* erythrocyte membrane protein 1, PfEMP1-based) vaccines only showed cross-reactivity to similar types of PfEMP1, but not for other types of PfEMP1 [56–58]. Therefore, it has been suggested that a combination of PfEMP1 antigen(s) and non-PfEMP1 antigen(s) is likely to be required to develop an effective vaccine [59], except in the case of VAR2CSA-based vaccines against pregnancy malaria (which are not covered in this review).

### Other antibody-based assays

Since EBA-175 region II is a binding region of the EBA-175 molecule to the erythrocyte, the blocking activity of human antibodies induced by a EBA-175 region II vaccine was tested using recombinant protein and erythrocytes, in addition to the regular GIA in a phase I trial [27]. When other EBAs and reticulocyte binding-like homologue (Rh) antigens reach to the clinical development stage, the erythrocyte binding assay might be used more frequently in human trials. However, whether the binding assay provides any additional information beyond that obtained from GIA, in terms of predicting vaccine efficacy, needs to be explored.

In case of ADCI assay, monocytes are utilized as the effector cells. On the other hand, the antibody-dependent respiratory burst assay utilizes polymorphonuclear neutrophils (PMN), and production of reactive oxygen species by the PMN is measured, rather than parasite killing [60]. The assay has not been utilized for any human trials, and similar to the ADCI, significant donor-to-donor variations of PMN sources are reported [61].

In case of anti-PfEMP1 antibodies, three more antibody-based assays have been utilized in animal immunization studies: an agglutination assay (i.e. whether a vaccine-induced antibody blocks agglutination of infected erythrocytes) [62], a rosette disruption (inhibition) assay [49,58], and a binding (or adhesion) inhibition assay [56,63–65]. Extensive studies have sought to identify the specific receptor(s) of each PfEMP1 antigen (domain) [59]; for example, a domain cassette 4-type of PfEMP1 mediates binding to intercellular adhesion molecule 1 (ICAM-1), and a VAR2CSA-type to chondroitin sulfate A (CSA). Therefore, in PfEMP1-based vaccine development, if a target molecule is known to mediate agglutination, rosetting, and/or binding either to a specific receptor (e.g. ICAM-1, CSA) or a specific cell type (e.g. endothelial cells), an inhibition assay using a vaccine-induced antibody can be applicable. However, since there is no common receptor or a common phenotype (agglutination or rosetting) for all PfEMP1 molecules, the assay should be tailored for each PfEMP1-based vaccine. Furthermore, a conserved epitope(s), which covers all diversity in PfEMP1 molecules, has not been identified. Those assays have been beneficial to understand the natural immunity in the field. However, considering the diversity of PfEMP1, it is arguable whether the assays are useful for development of a blood-stage vaccine that can actually reduce clinical malaria (or a specific type of clinical malaria, e.g. severe malaria) in the field. Since no PfEMP1-based vaccine (other than those based on VAR2CSA for placental malaria) has reached (or soon will reach) clinical development, it will take longer to evaluate the importance of those assays for vaccine development.

### T cell-based assays

Vaccine-induced T cell responses have been measured in multiple phase I trials with many different target antigens. The T cell-based assays include a proliferation assay (measuring proliferation of T cells against ex vivo immunogen stimulation) and measurements of immunogen-induced cytokine/chemokine (IFN-γ, IL-2, TNF-α, etc.) production by various methods (e.g. ELISPOT, intracellular cytokine staining, and ELISA) [66–70]. The accumulated data clearly show that blood-stage vaccines can induce T cell responses in humans. However, there is no strong evidence in humans that such T cell immunities induced by the blood-stage vaccinations work as an independent effector mechanism of protection (i.e. T cells by themselves or cytokine/chemokine released from the T cells directly kill blood-stage malaria) rather than to support antibody production and maintenance. If there is no independent mechanism, it is natural to assume that an antibody-based assay has a higher likelihood to be a surrogate than a T cell-based assay. In one human immunization study, four volunteers were inoculated with a low dose of *P. falciparum*–infected erythrocytes and drug cured three times, then challenged again with the same *P. falciparum*–infected erythrocytes [71]. Since the investigators did not find anti-malarial antibodies in the volunteers, the results from this immunization study suggested that T cell immunity worked independently. However, a later study revealed that the level of residual drug was unexpectedly high at the time of the last parasite challenge [72]. The results from the latter study made the interpretation of results from the former study difficult.

### Monkey challenge model

The monkey *P. falciparum* challenge model has been a useful tool to evaluate many different vaccine formulations and also has been used to find novel candidates [34,73–77]. Many monkey studies were conducted with Freund’s adjuvant to induce maximum immune responses, but Freund’s adjuvant cannot be injected into humans because of its toxicity. To make this model more valuable, several studies were conducted with human-applicable adjuvants or vaccine formulations, and showed protective effects in some studies [34,75,77]. Monkey challenge studies can be done with non-GMP grade vaccines, and in contrast to the human challenge model (described next), investigators can follow the animals for a longer time until the monkeys develop high parasitemia or anemia. Therefore, the monkey challenge model might be a better model to evaluate the immunity against clinical disease, more than anti-infection immunity. For the negative side, because of the restriction in animal numbers which can be used for vaccine development and growing ethical concerns for using non-human primates, it has been becoming difficult to perform the monkey challenge studies in many countries. Another limitation is that since *Aotus* or *Saimiri* monkeys are not natural hosts of *P. falciparum* parasites, only a handful of strains (e.g. FVO, FCH/4, FUP-SP) which are adapted to the monkeys can be used for the challenge. Extensive discussions of the monkey challenge model have been published elsewhere [78,79].

### Controlled human malaria infection

The sporozoite challenge model has been broadly used for pre-erythrocytic vaccines. Since a pre-erythrocytic vaccine is designed to kill parasites before merozoite-stage parasites enter the blood stream, the sporozoite challenge model is an excellent model to evaluate efficacy. The same model has been applied to the combination of blood-stage and pre-erythrocytic stage vaccines [80–84], and also for pure blood-stage vaccines [68,85]. Other blood-stage vaccine trials involved blood-stage parasite challenges, instead of sporozoite challenges [33,86]. Several reviews have already described the difference between sporozoite challenge and blood-stage challenge [87–89], and the blood-stage challenge is considered to be a more suitable model for blood-stage vaccines. In both challenge models, participants need to be treated when the level of parasitemia becomes microscopically detectable (or earlier if a volunteer shows any symptoms). Therefore, in addition to the time to detectable parasitemia by smear, PMR (or parasite growth rate) are calculated in many trials to evaluate the vaccine effect more comprehensively [33,68,83,86]. There was a significant difference in median PMR between Gambian (2.4-fold/48 h) and UK (8.0-fold/48 h) adults, which indicates that if a vaccine can induce immunity such as seen in African adults, it may show significant reduction in PMR. One obvious limitation of this model is that the test vaccine needs to be safe and clear all regulatory and ethical standards before performing the challenge study, that is, we cannot use the model in preclinical trials. Another drawback was that only NF54 or 3D7 strains of parasites have been sufficiently standardized for inoculation into humans. To overcome this limitation, several groups have been working to expand the diversity of parasite challenges [90,91]. To date the human challenge model is considered to be the closest to a phase II trial, but several questions need to be resolved in the future: (1) whether we should make a Go or No-Go decision for a phase II trial based on the CHMI results and (2) how much reduction in PMR is required to show efficacy in the field.

### Humanized mouse model

Since only limited laboratories can perform monkey or human challenge studies, and both of them are very expensive, testing the efficacy of blood-stage vaccines in a humanized mouse is one of the attractive alternatives if applicable. While significant progress has been made in the last 5–10 years [92], still a majority of the humanized mouse models require daily injection of human erythrocytes [93]. A study with a new humanized model where the mice were infused with human hematopoietic stem cells, instead of mature human erythrocytes, has been published [94]. While the mice could maintain human erythrocytes in their peripheral blood up to 4 months without daily injection, the level of human erythrocytes was <1% of total RBC (i.e. >99% of RBC were mouse erythrocytes) and the parasitemia in the total blood was only 3–5 parasites/μl. Since the humanized mice are basically immunodeficient mice, the mouse model could be used only for passive transfer experiments until recently. Huang et al. have reported very recently that they successfully reconstituted human CD4+ T and B cell responses in transgenic mice, in which a Pf-CSP vaccine could elicit protective immunity against a challenge with chimeric sporozoites (rodent parasites expressing Pf-CSP) [95]. However, further improvements are required to completely reproduce immune responses to a vaccine in humans. As more improvements in the humanized mouse model occur, this model will be used more broadly for future blood-stage vaccine development.

## Vaccine candidates that showed efficacy in humans

In this section, blood-stage vaccines that were reported to show some efficacy in human clinical trials or CHMI models are discussed, and the summary of those trials are presented in Table 1. All of the antigens described in this section are merozoite surface antigens or antigens secreted from merozoite, and they are considered to have critical roles during merozoite attachment and/or invasion of erythrocytes.

**Table 1.** Blood-stage vaccine candidates that showed significant effects in humans.^a^

Trial	Vaccine formulation	Main outcome	Ref
*Combination B (MSP1, MSP2, and RESA)*			
Phase IIb	*E. coli* expressed recombinant MSP1 (K1 strain), MSP2 (3D7), and RESA (FCQ-27/PNG) proteins with Montanide ISA720 adjuvant	No clinical protection, but strain-specific reduction in malaria infection: 78 out of 359 (22%) PCR samples showed 3D7 dimorphic form of MSP2 in the control groups, while 30/360 (8%) in the vaccine groups	[96]
*AMA1*			
Phase IIb	*E. coli* expressed recombinant AMA1 (3D7) protein with GSK AS02_A_ adjuvant	No clinical protection, but strain-specific reduction in malaria cases. In 22 episodes (out of 271 total episodes observed during the trial) infected with AMA1-3D7 type parasites, 16 cases occurred in the control group, and another 6 cases in the vaccine group.	[97]
Phase IIa	*E. coli* expressed recombinant AMA1 (3D7) protein with GSK AS02_A_ adjuvant	No significant difference in prepatent period or parasite growth rate after sporozoite challenge. However, significantly lower cumulative parasitemia during Day 7–9 after challenge in the vaccine group (*n* = 10) as compared to the unvaccinated infectivity control (*n* = 6).	[68]^b^
*MSP3*			
Phase Ib	MSP3 long synthetic peptide with aluminum hydroxide adjuvant	Significant reduction in risk of clinical malaria: 1.2 (15 μg dose) and 1.9 (30 μg dose) cases per 100 days per person in the vaccine groups (*n* = 15 each) while 5.3 in the control group (*n* = 15)	[98]
*SE36 (SERA-5)*			
Phase Ib	*E. coli* expressed recombinant SERA-5 (Honduras-1) protein with aluminum hydroxide adjuvant	Significant reduction in risk of clinical malaria: Hazard ratio = 0.26 after adjustment of age and gender: the vaccine group (*n* = 66) was compared to the control group (*n* = 16) and newly enrolled unvaccinated individuals (*n* = 50)	[99]
*MSP1*			
Phase IIa	Recombinant chimpanzee adenovirus 63 (ChAd63) and modified vaccinia virus Ankara (MVA) vectors encoding MSP1	Significant difference in prepatent period after sporozoite challenge (*n* = 3 in the vaccine group and *n* = 6 in control) in the initial study, but not in the second study (*n* = 9 in vaccine and *n* = 6 in control)	[85]

^a^Clinical trials which were conducted with a multistage vaccine(s) are not included.

^b^There were other vaccine groups in the trial, but only one group which showed a significant effect is shown.

### Combination B

Combination B vaccine contained MSP1 (K1 allele), MSP2 (3D7 allele), and RESA (FCQ-27/PNG allele) antigens and was formulated with Montanide ISA720 adjuvant in a phase II trial with 120 children (5–9 years old) [96]. The 120 individuals were first divided into two groups; children were given either sulfadoxine-pyrimethamine (SP) or a placebo 1 week before immunization. In each group (SP or non-SP), half of the children received the Combination B vaccine and the other half got placebo vaccine (adjuvant alone); then parasite density and clinical malaria were monitored. Among the SP group, there was no effect on parasite density. In contrast within the non-SP group, the vaccine significantly reduced parasite density (*p* = 0.024). While there was a ‘mathematically’ significant reduction in parasitemia in the SP group, the vaccine did not show a ‘biologically’ significant effect in both groups, viz. no efficacy against clinical malaria. When both SP and non-SP groups were combined, children who received Combination B vaccine had less chance to be infected with parasites with the 3D7 form of MSP2: 78 out of 359 (22%) PCR samples collected from placebo group, and 30 out of 360 (8%) from vaccine groups (*p* = 0.04). The number of participants in each group was small (*n* = 30 each). This is the first blood-stage vaccine showing an allele-specific effect in malaria-exposed children. While the safety and immunogenicity results of a phase I study with MSP2-C1 (combination of 3D7 and FC27 allelic forms of MSP2) was reported in 2011 [40], according to the WHO malaria rainbow table [100] and ClinicalTrials.gov [101], no human trials with Combination B vaccine or other MSP2-based or RESA-based vaccines are planned in the near future.

### AMA1

While many phase I trials have been conducted with AMA1 vaccines, only two phase II trials have been completed so far. One of the studies involved AMA1-3D7 protein adjuvanted with AS02_A_ (FMP2.1/AS02_A_) and was tested in 1–6 year Malian children [97]. A total of 383 children were followed completely for 240 days. Similar to the phase II trial with Combination B vaccine, there was no significant impact on clinical malaria. However, when only children who were infected with AMA1-3D7 type parasites (determined by cluster 1 loop of domain I sequences, the most polymorphic region in AMA1 molecule) were analyzed (22 episodes out of 271 episodes observed in the trial), there was a significant effect by the AMA1 vaccine: out of the 22 clinical episodes, 16 occurred in the control group and 6 in the vaccine group (*p* = 0.03). The follow-up study showed no strain-specific protection in the next year [102]. Another phase II trial with a mixture of AMA1-3D7 and AMA1-FVO formulated on Alhydrogel in 2- to 3-year-old children (279 children were followed completely for 154 days) showed no impact on clinical malaria [103] or strain-specific protection [104].

In two human homologous sporozoite challenge trials, small, but significant, effects were observed in terms of cumulative parasitemia [68] or PMR [83]. However, neither study showed significant delay in treatment time (i.e. time to reach a level of parasitemia detected by light microscopy).

AMA1 is a highly polymorphic protein, and the phase II trial with FMP2.1/AS02_A_ showed allele-specific protection. The GIA result from a phase I study indicated that a mixture of AMA1-3D7 and AMA1-FVO is unlikely to cover the variations in the field [105]. Therefore, many investigators have been trying to overcome the polymorphic issues for future AMA1-based vaccines. In animal immunization models, mixtures of 4 or 5 AMA1 proteins [106–108] could induce strain-transcending antibodies as judged by GIA. A rabbit study indicated the possibility that a modification of immunization strategy may further improve the cross-reactivity (i.e., immunize different AMA1 proteins sequentially, rather than inject the mixture of proteins every time) [109]. Another unique approach is to generate chimeric AMA1 proteins (DiCo) which could cover the majority of polymorphisms in the field by combining 3 DiCo proteins. The results of a non-human primate study were promising [110], and a phase I trial is underway (ClinicalTrial.gov Identifier NCT02014727). One more unique approach is to mutate major polymorphic residues to alanine, glycine, or serine [111]. While the chimeric AMA1 induced more cross-reactive antibodies judged by GIA, the levels of inhibition were lower compared to the antibodies raised with non-chimeric AMA1 for the same strains of parasites.

Researchers also have been attempting to generate more potent AMA1-based vaccines. AMA1 and rhoptry neck protein (RON) 2, 4, and 5 form a complex during merozoite invasion [112]. A recent animal immunization study suggests co-injection of AMA1 and RON2 may improve the efficacy of responses to homologous parasites [113]. Further study is required to determine whether the enhancement occurs in humans and whether the strategy works with AMA1-mixtures or DiCo vaccines.

### MSP3 and GMZ2

MSP3-based vaccines could induce protective immunity in a monkey challenge model [114]. In addition, vaccine-induced human anti-MSP3 antibodies showed ADCI activity *in vitro* and killed *P. falciparum* parasites injected to humanized SCID mice in the presence of human monocytes [41,115]. While the efficacy was not tested in a phase II trial, efficacy was reported from a phase Ib trial in 1- to 2-year-old children conducted in Burkina Faso [98]. In the trial, groups of 15 children received either 15 μg of MSP3 long synthetic peptide (MSP3-LSP), 30 μg of MSP3-LSP, or control hepatitis B vaccine. The incident rates of clinical malaria in the MSP3 groups were significantly lower than that in control group (1.2 cases per 100 days for 15 μg MSP3, 1.9 for 30 μg MSP3, and 5.3 for control, *p* = 0.01). Another vaccine, GMZ2, which contains MSP3 and GLURP is also under investigation [116–119]. The GLURP itself induced ADCI active antibodies in humans [42]. According to the WHO malaria rainbow table [100], both MSP3 and GMZ2 are the only two blood-stage candidates under phase IIb evaluation (no information in ClinicalTrials.gov [101]).

### SE36

SE36 vaccine contains a part of SERA-5, results from one phase Ia trial [120] and from one phase Ib trial in Uganda [99] have been reported. In a part of phase Ib trial, 66 individuals (6–20 years old) received SE36 vaccine and were followed for clinical malaria episodes between 130 and 365 days post-second vaccination. In addition to the 16 individuals who were enrolled from the beginning and received saline (instead of SE36), the investigators newly enrolled 50 individuals before the clinical follow-up as the control group (*n* = 66 total) to increase the power of the study. After adjustment for age and gender, the risk of parasitemia ≥ 5000 parasites/μl plus fever was significantly lower in the SE36 group compared to the control group (hazard ratio = 0.26 (95% CI, 0.10–0.61); *p* < 0.01). While there is no plan for a phase II trial (according to WHO malaria rainbow table [100] and ClinicalTrials.gov [101]), the efficacy of SE36 ought to be confirmed by a phase II trial.

### MSP1

Multiple monkey challenge models showed MSP1-based vaccines could induce protective immunity in monkeys [35,121,122], and MSP1 is one of the components of Combination B vaccine as discussed above [96]. Only one phase II trial with pure MSP1-based vaccine has been conducted until today. The phase II trial conducted with MSP1_42_-3D7 vaccine adjuvanted using AS02 in Kenyan children showed no significant effect on clinical malaria [123]. The significant effect of a MSP1 vaccine was only observed in one phase IIa trial where adults were immunized with chimpanzee adenovirus 63 (ChAd63) followed by modified vaccinia virus Ankara (MVA) [85]. While the number of vaccinees was very small (*n* = 3 in the vaccine group, and *n* = 6 in control), there was a significant delay to the time to diagnosis by microscopy (*p* = 0.035) after sporozoite challenge. However, when the same ChAd63/MVA vaccine was tested in another phase IIa trial (*n* = 9 for a vaccine group and n = 6 in control), the MSP1 vaccine showed no significant effect (*p* = 0.13) [85].

It is likely that a stronger adjuvant and a new immunization strategy are required to make an efficacious MSP1-based vaccine.

### Vaccines against multistage parasite antigens

NYVAC-PF7 is an attenuated vaccinia virus containing genes encoding candidates from multiple stages: pre-erythrocytic (CSP, SSP, and LSA1), blood-stage (MSP1, AMA1, and SERA), and mosquito-stage (Pfs25) antigens. When the volunteers were challenged with sporozoites (*n* = 35 in two doses of vaccine groups and *n* = 8 in the control group), there was a significant delay in prepatent period [81]. PMR was not measure in the trial. A significant effect by another multistage vaccine was also reported. PEV3A vaccine included peptides from CSP (pre-erythrocytic) and AMA1 (blood-stage) [83]. While there was no significant difference in prepatent period, PMR in PEV3A vaccinated group (*n* = 5, 5.7 parasites per ml per cycle) was significantly lower than that in the control (*n* = 5, 8.7 parasites per ml per cycle). However, since vaccines which contained only pre-erythrocytic antigens or blood-stage antigens were not tested in both studies, it is practically impossible to estimate how much (or any) protective effects were elicited by the blood-stage antigens.

## Other vaccine candidates under clinical development

### EBA175

A cysteine-rich second region of EBA-175 (EBA-175-RII) vaccine has been tested in a phase Ia trial, and the vaccine induced GIA-active antibodies. In a recent rabbit study, antibodies against more conserved regions of EBA-175 (regions III–V) showed stronger and more strain-transcending activities judged by GIA [124], while the functional activity of regions III–V is unknown. Further investigation is required to reveal whether the region III–V vaccine is better than the RII vaccine in humans. Since different field parasites show different protein expression levels of EBA and Rh proteins (EBA-140, EBA-175, EBA-181, RH1, and RH2 were tested in the study) [125], all of which are involved in redundant merozoite invasion pathways, a combination with other antigen(s) is likely to be required to show efficacy in the field.

### P27A

P27A is a part of Trophozoite exported protein 1 (Tex1, previously called hypothetical protein PFF0165C). The Tex1 antigen was found by a unique approach, that is, based on the α-helical coiled coil structure of the molecule [126]. In contrast to other blood-stage candidates, Tex1 is not a merozoite protein, and locates at Maurer’s clefts in infected erythrocytes [127]. Human affinity-purified P27A-specific antibody and rabbit anti-P27A antibody showed ADCI activities [126,128], and the first phase Ia and Ib trial with Alhydrogel or GLA-SE adjuvants was completed in July 2015 (ClinicalTrials.gov Identifier NCT01949909). Several other antigens tested in the structure-based screening study showed ADCI activities [126], and they are also interesting candidates for further investigation.

### RH5

RH5 is one of the reticulocyte binding-like homologue (Rh) proteins, and an RH5 vaccine could induce strain-transcending antibodies in animals judged by GIA [129]. In addition, while the antibody levels in malaria-exposed individuals are low compared to other merozoite antigens [129,130], affinity-purified human anti-RH5 IgGs also showed GIA activity [130,131]. The low immunogenicity in humans may explain the reason why there are very limited polymorphisms in the molecule [132]. In an *Aotus* monkey study where the monkeys were immunized with the 3D7 sequence of RH5 using human-compatible vaccine formulations (ChAd63 vaccination followed by either MVA boost, or recombinant RH5 protein-Abisco-100 adjuvant boost), the vaccine induced a protective effect against heterologous FVO parasite challenge [133]. A phase Ia trial with ChAd63-MVA vaccines is ongoing (ClinicalTrials.gov Identifier NCT02181088).

In addition to studies with RH5 as a stand-alone vaccine candidate, many animal immunization studies have been conducted using mixtures of vaccines including RH5. For example, *P. falciparum* RH5 interacting protein (PfRipr) [134] and cysteine-rich protective antigen [135], both of which make a complex with RH5 during the merozoite invasion, can induce GIA-active antibodies by themselves and in combination with RH5. Furthermore, other merozoite antigens, such as EBA-175, RH1, RH2, RH4, AARP (apical asparagine-rich protein) and Pf38, have also been evaluated with RH5 [131,134–137], and the vaccines induced cross-reactive functional antibodies judged by GIA. Of interest, some combinations of IgGs showed synergistic invasion inhibition in GIA [135–137]. At this moment, it is not clear whether such synergistic protective effects can be observed *in vivo*.

## Other vaccine candidates

In addition to the candidate antigens described above, many more potential candidates have been proposed from longitudinal cohort studies [14,138,139]. *P. falciparum* schizont egress antigen-1 (PfSEA-1) is one of the novel candidates found from cohort studies [140]. Similar to the P27A (Tex1), PfSEA-1 is not a merozoite antigen and localizes at the parasitophorous vacuole membrane, Maurer’s clefts, and the inner leaflet of the erythrocyte membrane. The anti-PfSEA-1 antibody prevents parasite egress rather than merozoite invasion. Anti-Pf332 antibody is likely to work with a similar mechanism [141], while a recent study suggested it might also block parasite growth through a different mechanism [142]. The data from P27A, PfSEA, and Pf332 studies indicate that not only merozoite proteins or VSAs but also antigens expressed within infected erythrocytes could be targets of blood-stage vaccines. Many other candidates, which are not described in this review, are also known to induce GIA-active antibodies at least in animal immunization studies, such as MSP4 [143], EBA140 [144], RON3 [144], GAMA (glycosylphosphatidylinositol – anchored micronemal antigen) [145], EBL-1 (erythrocyte-binding ligand-1) [146], MSPDBL1 and 2 (merozoite surface protein duffy binding-like protein) [147,148], and RALP1 (leucine zipper-like protein 1) [149].

Several clinical trials have already been conducted with a mixture of multiple antigens (multiple stages) in different platforms, for example, AMA1+MSP1 [85,150], MSP1+EBA175 [151], CSP+AMA1 [82,84,152], and CSP+MSP2 [80]. Results from other multistage vaccines in animal immunization studies are also promising [153–155]. More multistage or multi-antigen vaccines are likely to be explored in the future. However, caution should be taken in such vaccines because antigenic competition has been observed in non-human primates and humans with AMA1+MSP1 vaccines [74,85]; that is, the mixture vaccine induced lower titers compared to the single antigen vaccine tested in the same study.

While it has not reached to the clinical trial stage, a group of researchers are investigating the possibility of using chemically attenuated blood-stage parasites as a vaccine [156], as a promising protective effect was observed by intravenous inoculation of attenuated sporozoites in humans in the case of a pre-erythrocytic vaccine [157].

## Expert commentary

There are several considerations to accelerate future blood-stage vaccine development. As mentioned above, none of the assays/models has been proven as a surrogate of protection and no blood-stage vaccines have shown strong efficacy in a large phase II (or III) trial. Therefore, novel antigen discovery should be continued with any approach available (e.g. identify immune-correlates with clinical malaria in a longitudinal cohort study, structure/sequence-based predictions). However, since resources are limited, the functional activity of antibody (and cellular immunity if applicable) against the novel antigen should be evaluated promptly using an assay/model with live human parasites (or transgenic parasites expressing the human antigen). If the novel antigen induces only a weaker activity than an existing candidate(s), the novel candidate may have a lesser chance to be a successful vaccine, unless it can induce a synergistic effect with other candidates. If no robust functional activity of the novel vaccine-induced antibody is detected by an *in vitro* assay, the investigators should consider the risk that vaccine development with the target molecule will be extremely challenging; viz. one needs to down-select vaccine formulations/adjuvants etc. without a reliable decision-making tool in the preclinical development stage and phase I trials. With new candidates which can induce functional antibodies and/or protection in a challenge model, a molecule that is known to be polymorphic and/or functionally redundant should be graded lower than a molecule which is non (or less)-polymorphic and/or functionally nonredundant, except when there is a methodology to overcome the issue.

Based on the published human trial data, the level of antigen-specific antibody which can be elicited by a vaccine in humans is expected to be somewhere between ~50 and several hundred μg/ml at the peak (i.e. 2–4 weeks after the final immunization), regardless of population immunized (malaria naive or immune, children or adults), antigen (e.g. AMA1, MSP1, RTS,S), adjuvant (e.g. AS01, AS02, CpG), or vaccine platform (e.g. recombinant protein, ChAd63/MVI) [26,68,70,102,105,123,150,158–160]. Without a major breakthrough in vaccinology (e.g. a completely new strategy, a new class of adjuvant), one of the crucial aspects in vaccine development is to use the several hundred μg/ml of antibody efficiently. Therefore, I feel a polymorphic and/or functionally redundant molecule has a lesser chance to be a successful vaccine unless the novel candidate can induce a strong parasite killing effect at very low concentrations of antibody. In line with this consideration, novel vaccines that only contain critical epitopes ought to be investigated further. Using functional monoclonal antibodies, chimeric antigens and other methodologies, researchers have tried to identify critical epitopes in several existing candidate molecules [43,106,108,161–163]. Since the highest limit of antigen-specific antibody concentration is likely to be set, that is, a few hundred μg/ml (unless there will be a major break-through), it is also important to determine vaccine-induced antibody level in a μg/ml-scale, rather than ‘antibody titer’ or ‘antibody units’, in a human trial. By doing that, the investigator could estimate whether there is any room to improve the immunogenicity (e.g. change immunization schedule and/or adjuvant to reach the few hundred μg/ml level) or consider switching to a new candidate/strategy.

One of the other important keys for the vaccine development is to increase capacity for performing phase IIb (and IIa) trials and test a promising candidate in humans as soon as possible. Many blood-stage candidates have been shown to induce functional antibodies in animals, and human affinity purified IgGs also have shown functional activities judged by GIA, ADCI assay, or other assays. In addition, some vaccines can induce protective immunity in monkey challenge models. However, significant efficacies of blood-stage vaccines have been observed only in small phase I or IIa clinical trials (or in small subsets of phase IIb trials). Therefore, we should prove or disprove the vaccine efficacy in a phase IIa or IIb trial as quickly as possible rather than spending a great deal of time and effort in animal and preclinical studies once a promising candidate (or a combination of candidates) is identified. The results from phase II trials provide strong feedback for further vaccine development, that is, for Go and No-Go decisions, which assay(s) should be used, and what level of (functional) activity needs to be reached.

Not only the peak immune response, the longevity of responses is likely to determine the vaccine efficacy in the field. However, at this moment, there is no universally accepted strategy which maintains ‘vaccine-induced immunity’ for a long time, and which ‘vaccine-induced immunity’ should be measured as the surrogate of ‘protection’. A further complication is that there is no consensus on the best indicator of ‘protection’ in a phase IIa and IIb trials with a blood-stage vaccine. In epidemiology studies, different (or multiple) measurements have been reported (e.g. time to first malaria episode, risk of clinical cases per time per person). The selection of the ‘clinical protection’ readout(s) needs to be determined based on a target product profile of a vaccine, but if we aim to make ‘vaccines with >75% efficacy against clinical malaria (The Malaria Vaccine Technology Roadmap [10])’, the vaccine should show a significant effect in any measurements.

## Five-year view

I expect results from AMA1-DiCo, GMZ2, SE36, MSP3, P27A, and RH5 clinical trials will be available in the near future, and the data will guide further blood-stage vaccine development. Several other novel candidates described above may reach phase I or IIa trials in the next 5 years. In addition, it is likely that more development efforts will be focused on multi-antigen and multistage vaccines. Not only such ‘broader’ approach but also a ‘deeper’ approach (i.e. epitope specific approach) for each target antigen is also anticipated. For the host side, whole transcriptional analysis to identify biomarkers of protection is being applied to a pre-erythrocytic vaccine [164]. Once a blood-stage vaccine shows clear efficacy in a phase IIa (or IIb) trial, transcriptional analysis in the hosts will be explored further. In addition, further studies with human and humanized monoclonal antibodies (e.g. a passive transfer study with these antibodies in a CHMI model) will be conducted to explore the interaction with the parasites in the human host.

## Key issues

- No *in vitro* assays or challenge models have been shown to be a surrogate of protection in the field for any blood-stage vaccines.
- However, GIA/IIA and ADCI assays are used as function assays in many preclinical and clinical studies.
- Monkey parasite challenge model has been a useful tool to evaluate blood-stage vaccines in preclinical stages.
- Parasite challenge model, especially blood-stage challenge, in phase IIa trials is valuable and is becoming more widely used.
- Only Combination B, AMA1, MSP3, and SE36 vaccines were reported to show detectable levels of efficacy against either total or allele-specific parasites in humans.
- However, the size of the clinical trials (or subsets of the population analyzed in the clinical trials) was less than 70 per arm. A larger trial is necessary to confirm these findings.
- Many potential novel candidates have been identified in the last 5–10 years, and several novel vaccines (P27A, RH5, etc.) are under clinical investigation.
- Multi-allele, multi-antigen, and/or multistage vaccines need to be investigated further.
